# An Analysis of Guislain's Work on Insanity

**Published:** 1853-07-01

**Authors:** 


### AN ANALYSIS OF GUISLAIN'S WORK ON INSANITY.

### Third Lecture.

*Of the Elements which must enter into the definition of mental diseases.*

### First Part.

*General Considerations.*—Presently I shall speak of the phenomena which characterize mental diseases; and I shall endeavour to present them to you in the patients who will be brought before you.

I shall, in the first place, consider the definition of mental diseases. In the next place, I shall enter upon the classification and symptomatology of these affections. I shall discuss the cadaveric phenomena, and as far as art permits, I will point out to you in the living patients the signs of the lesions found after death. I shall trace the etiology and the pathogeny. I shall analyse my registers in order to deduce the prognostic indications. I shall pass in review all the resources of treatment. Finally, I shall devote several lectures to the examination of the questions relating to the construction and organization of lunatic asylums.

*How a lunatic is recognised.*—It is in the first place important to determine the common character presented by all the inmates of this establishment. The collective feature is, pre-eminently, a remarkable change in their actions. Their conduct has no longer been what it was; their extravagancies have caused them to be observed in the populations which surrounded them. The man of former times has disappeared: he is replaced by a new man—a lunatic.

This state is a disease. But the most prominent phenomenon of disease—fever, is wanting.

*Moral Incapacity.*—There is observed among the insane an incapacity which is peculiar to them, a moral incapacity. The madman does not understand his interests, himself, or society. The *ego* is withdrawn from their thoughts and their actions. Remove these patients from this place, deprive them of the assistance of their families, of the security of the law, and the most deplorable lot awaits them. They will cease to appreciate the means of existence; they will be incapable of conducting their affairs; they will be disgusting from their filth; some will believe themselves rich, and will die of hunger; others will steal, commit arson, kill, without being aware that they are acting in opposition to laws, human and divine.

*Conscience, Moral liberty.*—Among all, the obscuration of certain faculties renders the examination which a man makes of his thoughts and actions difficult or impossible. In the sane man there is a mental mirror. He examines himself in this reflector, and exercises a judgment upon himself. That is his conscience. Well, the madman loses this attribute, he loses the faculty of self-knowledge; and what is more, he loses the power of self-control.

But you must not conclude that alienation excludes in all these patients the faculty of reasoning. There are madmen who acquire a dialectic, a logic faculty, a richness of ideas, forcibly contrasting with their normal condition. A patient who believes that poison is mixed with his food, starts from a sophism, but reasons perfectly well.

There are madmen in whom the entire sphere of ideas remain intact, and in whom the disorder exclusively affects the sentiments or the impulses.

There are madmen who, at the period of incubation or of the invasion of their disease, tell you they are ill, and that their fit is about to break out.

There are *suicidal* patients, who will sometimes entreat you to watch them, and control their movements.

It will occur to you to put the following questions to certain madmen: Why are these men here? The patient will reply, Because they have lost their senses.—And you? Oh! that is quite another thing, I am not mad.

There are cases in which a man preserves all his intelligence, and understands his position. But these cases are not very frequent; and usually it is only at the onset and at the decline of the morbid state that this is observed. If a man affected in this manner has the power of conducting himself properly, he may indeed be diseased in mind, but he is not insane in the proper acceptation of the word.

*Delirium and moral liberty.*—Here is a singular case: this patient has been four years in the asylum. He is affected with exaltation and perturbation of the ideas. Some months ago he expressed a strong desire to return to his family. I allowed him to go out walking accompanied by a servant: the change of impression had a most happy effect; he has acquired a gentleness of character; and I look upon him as convalescent, or even cured. But observe the singularity of this condition. This man answers in the best possible manner any question you may put to him; he tells you with evident conviction that he must employ violent efforts over himself to stop the singular words he pronounces, when he wishes to express an idea; he assures you that he is aware of the moment when he is about to utter nonsense; frequently he even succeeds in not articulating the words which he feels rising to his lips, and in some measure, in driving them back. But what is remarkable is, that as soon as you speak before him the odd words and phrases which he was in the habit of uttering, he relapses instantly into his primitive state, and his speech is henceforth nothing but a torrent of incoherent words. What is yet more remarkable, is that he comes *voluntarily* out of this situation, as you will see . . . . .

This man uses great precautions in his speech to avoid falling back into his delirious ideas. The remarkable thing in him is the effort he makes to preserve his reason.

Observe well, therefore: 1st. In alienation, all the mental faculties may be overthrown.

2nd. The madman may continue to understand everything but his own condition.

3rd. Conscience may remain unaffected, and the patient may say to himself: I am a madman.

4th. The faculty of creating this state, or of causing it to cease, is what the insane do not possess, unless they are convalescent, as you have just seen.

*Alienation—Infancy.*—This condition resembles infancy; but infancy in a state of exaggeration. Like infants, the insane are credulous, timid, and scarcely estimate the bearing or the consequence of their acts.

It is for this reason that in the eyes of the law, the madman is not responsible; he is classed in the category of minors.

*Alienation—Dreams — Somnambulism.* — Some authors have compared alienation to dreams. But if we consider that sleep, the suspension of sensations, muscular prostration exist during dreaming, and do not characterize mental alienation, we find in these two conditions a difference so great as to warn us not to seek for a very intimate relation.

Whosoever has closely witnessed the phenomena of somnambulism, will discover between this state and alienation a nearer analogy than between dreaming and insanity. In somnambulism, as in alienation, something has detached itself from the moral, intellectual man; the regulator of the phrenic operations is out of order; the reflecting mirror is, so to speak, covered with a veil. Nevertheless, there is this great distinction,—in somnambulism the patient sleeps, whilst the madman does not sleep when he is said to be awake.

### Second Part.

*How to arrive at the definition of mental alienation.*—We meet with extreme difficulties in finding common characters applicable to all the insane. There does not exist a single formula which can be considered as a classical definition.

Nothing is less easy than to answer the question: What is a madman?

Those who have occupied themselves with the description of mental diseases, have avoided the logical *résumé* of this condition. The reason is, that it is often difficult to say when disease of the understanding commences, and where moral health ceases.

This is the way to succeed in this operation:—We begin by placing in prominent relief all the great symptomatic characters of mental alienations; they may be reduced to the following heads:—

I. A manifestation contrasting with the acts and ideas of persons reputed sane, and with the habitual ideas and acts of the subject.
II. A congenital or occasional condition.
III. A condition regarded by scientific men as the expression of a morbid state.
IV. A chronic state.
V. An apyretic state.
VI. A state presenting a tendency to the production of periodical returns.
VII. A state entailing a more or less absolute impossibility of observing customary laws and usages.
VIII. A state entailing the impossibility of managing self or business.
IX. A state most frequently of irreflection.
X. A state always of irresistibility.
XI. A state always of irresponsibility.

Thus, reducing all these elementary phenomena to a more concrete formula, we say that mental alienation is:

A chronic apyretic disease in which the ideas and acts are under the dominion of an irresistible power; a change which has taken place in the manner of perceiving, conceiving, thinking, and acting; in the attributes of the character, in the habits; a state which contrasts with the sentiments, the thought, the acts which surround the individual; an affection which makes it impossible for him to act with a view to his preservation, to his responsibility, and to his obligations towards God and society.

This definition, as I have given it, errs by its diffuseness; we must still further condense the materials. We may then say:

*Mental alienation is a morbid, apyretic, chronic derangement of the mental*

*faculties, which deprives man of the power of thinking and acting freely with a view to his welfare and his responsibility.*

The man ceases to be free. The absence of liberty is what we meet with in every mental disease.

*Distinctions to be made.*—Some of the elements which enter into this definition are met with in all the kinds, in all the varieties of the phrenopathic state. These are :—

A.—The disease, without continuous fever.

B.—A disorder of some kind of the intellectual faculties.

C.—The insufficiency of these faculties, in reference to the wants, welfare, security, and responsibility of the individual.

In many cases it will prove a difficult task to distinguish these states, as regards the moral proofs, from the freaks, the caprices of a violent or eccentric character; from the moral, physiological, deeply-felt pain of the passions; from error; from a zeal carried to exaggeration; from vice and crime; from libertinism and depravity of appetite; from the thirst of greatness and riches; from contempt of life; from feebleness of intellect, and from many other conditions.

### Third Part.

*Conditions which must not be confounded with mental diseases.—The fool of society.*—How many frivolous men are there who attract the eyes of the crowd, and who, nevertheless, are not mad, although in ordinary life they are so designated! What oddities of costume, what whimsical fancies in building, in the arrangement of furniture!

The line of demarcation between wisdom and insanity is sometimes very difficult to define according to the rules of science, and yet the vulgar is rarely deceived. They discover the limit by instinct.

*Disturbers of public order.*—The absence of respect for the laws is not an indication of insanity, when it is manifested apart from other intellectual or moral derangements. The true reforming madman is a man who, besides his subversive ideas, betrays a disease of the intelligence, an impairment of the conception, an imagination creating absurdities.

The especial characteristic of mental alienation, when this does not consist in native imbecility, is its pathological character. Alienation has its prodromata, its phases of intercurrence, between which the normal state re-appears; it has also periods in which reason abdicates its throne. It has a propensity to spontaneous returns; it presents special changes in the state of the *primæ viæ*, in the pulse, in the locomotive movements.

There is, in a diagnostic point of view, a *criterion* sufficiently general, which M. Falret has well drawn: this is the change which takes place in the habits, ideas, acts, and gestures of the man who has become insane. This phenomenon cuts the knot of all the great questions, at a period when everything is still obscure in the appreciation of the disease: it is the comparison of a man with himself.

Frequently the chronicity of the condition calls attention, and puts the physician on the path of determining the disease. Natural grief, as for the loss of a relative, lasts for a time, and disappears. Not so with morbid grief: this grows and strengthens for months and years.

Anger rises in a moment, and is dissipated in a few minutes, hours, or days; the anger of alienation endures much longer, for years, and for life.

There is greater evidence, greater intensity, if I may so speak, in mental alienation than in passion, in error, or in simplicity. There is no sorrow like morbid melancholy, no anger like furious mania, no illusions like the conceptions of delirium, no weakness of mind like idiocy.

What is true with regard to the passions, is no longer so as regards certain exaltations. Thus, the passion of religion may last throughout life without being a mental alienation. To distinguish the devout man you must have other elements of comparison than the lapse of time.

*Religious martyrs.*—The cenobites of the cloisters, the Trappists, the martyrs—are these men in the possession of all the faculties of reason—men who consecrate themselves to a life of privations and continual punishments? Are not these men religious monomaniacs? No; the reason of these men does not differ from that of the masses in the midst of whom they live; the masses do not look upon them as insane. The chief of the corporation has the power of modifying the habits of the most austere devotee; he submits, obeys; acts with method, and in the sense of his obligations; if his chief makes an appeal to his zeal he bows to his will. The insane religionist, on the contrary, follows only his own aspirations, he listens to nothing, he changes nothing in his habits; he only obeys through caprice: his state is one of irresistibility.

*Debauchees.*—There are men and women who are insatiable with regard to sensual pleasures; are these insane, or are they only persons consumed by the fire of the passions? We meet some of these unfortunate beings who implore the aid of art, the help even of the priest, when the fulfilment of their wants does not calm their unwonted ardour. No; these are persons who are ill, and as they know how to govern themselves, must not be looked upon as insane; although related to the insane, they must be classed in the category of hysterical and other patients.

You will say that prostitutes should be comprised in the number of the insane. In the prostitute there is something different to mental disease. She ceases to offer herself when she is no longer sought for. But the erotic mad-woman does not perceive the decay of her charms; she always offers herself, and believes herself always young and beautiful.

*Suicidal persons.*—Suicide is a question about which our opinion must be formed with greater uncertainty. By many suicide is referred to a morbid condition; many regard it as a physiological act.

Physiological suicide, like murder, robbery, may be referred directly to certain causes. Good advice, reflection, religion, an error discovered, modify the resolution of the man who commits them. With the madman, suicide is an irresistible act; it has its precursory and its concomitant phenomena. Besides the principal phenomenon we discover disease; it does not depend upon the individual to arrest it.

*Misers, Thieves, Murderers.*—Will you say that that miser is insane who dwells in an infected atmosphere, who, perishing with cold in the winter, lives upon bread, and who is found after his death to have concealed an unexpected treasure? He is, indeed, a monstrosity, but he cannot be said to be of diseased mind. His passion is a vice of character and not a disease; the symptoms and the course of disease are wanting, viz., the invasion, the remission, the periodicity.

It is the same with what is called in society the monomania of theft. If it were made law that misers, at a certain point, should undergo punishment, they would be seen, like the self-called kleptomonomaniacs, to modify their acts under the influence of the rigours of a prison.

But we must own that there are doubtful situations in which it is more reasonable to detect a mental aberration than a crime. The most difficult cases are those in which a natural weakness of intellect imparts to acts a preponderance which breaks the equilibrium between the characters of animality and those which belong to man.

The entire history, the life of the accused must elucidate the question. The brutalization of the individual, the original defect of his intellect, may not have permitted him to judge of his inclinations and his acts with the proper force of reason.

Criminal history has of late furnished material for many a controversy.

Georget was the first to relate facts and direct attention to this subject. We must not dissemble the difficulty of the question raised by these facts when irresistibility is to be proved. But to a practised mind, especially when the subject whose life is to be investigated can be observed for a certain time, difficulties vanish; in the end we discover the sum of the characters whether of health or of disease. The impairment of the faculty of self-examination, the impossibility of understanding his position, a certain oscillation, and other phenomena, reveal the morbid disorder.

*Visionaries.*—If we regard the question in the light of the reasoning power, the distinction between a man professing errors and a madman affected with delirious ideas may present a difficulty.

That which belongs to the sane man is a consciousness, the conception of having false ideas; when a man experiences visions, and tells you: "I see upon the wall grotesque figures, angels and demons, give me a remedy, something to eat," that will pass away, it depends upon debility; this person is not insane, inasmuch as he appreciates these phantasmagoria. But he might experience, at the same time, morbid inclinations which he was unable to resist, and in that sense he might be diseased in mind.

So long as the patient preserves the consciousness of his acts, and at the same time his free will, he is not insane, although he may experience a disorder in his ideas.

### Fourth Lecture.

### First Part.

*Of the necessity of reforming the vocabulary of mental affections.*—Nothing is so vague as the expressions employed to describe the intellectual phenomena. The words, moral intellect, understanding, reason, mind, mental condition, psychical condition, and many others require to be defined. The names, madman, insane, imbecile, maniac, are common terms, used to indicate general phenomena, whereas each ought only to serve as indicating a special phenomenon.

The languages of the North are not more clear in this respect than those of the South. Nor is there anything more incorrect than the Greek and Latin terms, created by the moderns, in their relation to the classes, genera, families and species of diseases. The greater number reveal the absurdity of the notions which prompted them. All have had in their origin a general signification; all partake of the infancy of art; all in the beginning designated a disordered reason. Some of them have become specialized as science has progressed.

The most ancient terms are: *Mania*, Melancholia, Insania, Dementia, Delirium.

Daremberg, the translator of the works of Hippocrates, says that the word *Mania* denoted among the Greeks a violent delirium, whilst Galen, according to Foes, uses it in the sense of melancholia, or chronic delirium.

The moderns, as Pinel, in his *Traité sur la Manie*, have often employed this term in a general sense, and by no means as implying violence, anger, fury. Esquirol has introduced the word into the special designation he invented to indicate melancholy, viz., *Lypemania.* Constituting mania a special phenomenon, he makes out of it his *monomania*, in order to define the partial delirium described by his predecessors. It is thus that you will find mania in *demonomania*, whilst terror should have been indicated, and not the exaltation of a maniac. It is for this reason that I say *demonophobia*, for the purpose of showing that this melancholy has for its fundamental element the fear of the demon.

*Melancholia* was first employed by the Greeks. It hardly suits our mental affections; it comes from *melas*, black, and *cholé*, bile. According to an aphorism of Hippocrates, the displacements of the peccant matter are dangerous in melancholy; they announce mania, blindness, spasms, or apoplexy.

The Romans often translated *mania* by *insania.* The aphorism of Hippocrates, relating to hemorrhoids and mania, is rendered: "*Si varices aut hæmorrhoïdes supervenerint, insaniæ solutio fit.*" They usually employed the terms: *insania, insanus, insanientes;* and thence, *l'insensé, l'insanité* of the French, and the *insanity* of the English. The terms *vesania, vesanus,* from *ve,* privation, and *sanus,* is also a derivative of *insanus* or *insania.*

*Delirium* is a word which is traced back to a very remote period. It has rarely signified a special mental alienation. It has been said to be derived from *Lira,* a furrow drawn in a straight line; this would mean in our present language, a mind that is warped.

*Dementia, de,* privatus, and *mens, menos,* mind, soul: this term is very old, and indicates very exactly that condition in which the mental forces are defective. Hence the *dementia,* and also the *amentia* of modern pathologists. *Vecordia* has an almost analogous signification.

Our codes speak only of mania (fureur), and of the maniacal (furieux), of imbecility, of dementia, and of the insane. Nowhere will you meet the expression, melancholy or melancholic, and yet morbid sorrow, from its extreme frequency, must have attracted the attention of legislators. But those who have made our laws, have reproduced the ancient ideas of legislation, and as formerly nothing was seen in morbid sorrow but a vice of the humours, we understand why it is that they have excluded melancholy from the number of mental diseases.

Confusion of words always announces confusion of ideas. It is so in the diseases which engage our attention; for I know not one kind of mental affection which is not badly defined by the term which is used to indicate it. Unfortunately these terms are consecrated by the law, and for that reason they may lead to the most deplorable judgments.

The vulgar words, *mad, madness* (*fou, folie*), have taken place in the vocabulary of science since French authors have ceased to write in Latin.

*Mental alienation* is modern, at least, as regards the French expression, for *alienatio mentis* was in use in Rome. The word is found in Sauvages. The Germans have derived from it their *Seelenstörungen,* disorders, troubles of the soul. The Germans have mostly seen in mental diseases a disorder of the senses: hence their *Wahnsinn,* Blödsinn.

The Italians have most frequently conformed to Latin words. But they have *pazzi, pazzia, pazzarelli,* which call to mind the words *folie* and *fous* of the French.

### Vocabulary.

Science demands precision, and consequently the adoption of a radical term, considered in a general acceptation. This term must express a disease distinct from those affections with which it might be confounded, and it must be medical.

*Kephalê* cannot supply it: it is not diseases of the encephalon, of the head, which we have to designate; but functional affections of the domain of the ideas, sentiments and passions. This radical term I find in *Phren*—this word comprises the intellectual acts proper to man. In every case, *mental* is preferable to *psychical: Mens* is very clear; *Psyche* is not at all so. In some times past, especially in Germany, Psyche has been employed for the formation of the terms relating to mental diseases. Thus we have Psychology, Psychiatry, Psychoses, and Psychopathics. I prefer the substantive Phren, for the following reasons:—Phren is a designation that is understood; it dates from the Hippocratic era. We find it in *Phrenitis,* a word met with in the works of the Father of Medicine, in those of his disciples, and in Celsus, as well as *Paraphrenitis,* by which the Greeks distinguished acute delirium. The idea of connecting this delirium to cerebral inflammation belongs to *Ætius*. *Phrenology*, *phrenological*, we owe to modern times. *Psyche* is more philosophical, at least more theological, in the sense attached to it by St Paul. Moreover the word *Phren* is more pleasing to the ear; it lends itself admirably to the formation of new words.

Thus, from *Phren*, *phrenis*, I make

*Phrênie*, *frenie*, mental state in the largest sense.

*Phrenic*, what belongs to the *Phren*.

*Phrenography*, a discourse which treats of the *Phren*, the intellectual and moral qualities of psychology.

*Phrenology*, science of the phenomena of the understanding, already employed by Spurzheim.

*Phrenologue*.

*Phrenological*.

*Phrenopathist*, a mental physician.

*Phrenocomium*, a lunatic asylum.

*Phrenotyrb*, disorders of the intellectual, moral functions: *Scelenstörung*.

*Phrenopathy*, mental disease, psychosis.

*Phrenopathic*, a madman.

*Phrenotherapeia*, mental therapeutics, psychiatry.

*Phrenalgia*, moral grief, melancholy, lypemania.

*Hyperphreny*, hyperphrenopathy; exaltation of intellectual acts; passions, mania.

*Paraphreny*, beyond exaltation, eccentricity, insanity.

*Phrenoplexy*, moral commotion, ecstasy.

*Ideophreny*, delirium.

*Aphreny*, absence of moral or intellectual faculties.

*Phrenotrophy*, it is thus that Fuchs names idiotcy.

*Phrenesy*, frensy; inflammation of the brain, of the meninges, acute, accompanied by violence.

*Phrenetis*.

*Orthophreny*, moral intellectual education.

The above substantive words have their corresponding adjectives.

I shall preserve, by preference, the received denominations, melancholy, mania, insanity; but I shall employ them in a definite sense; they will serve to specify the elementary genera. Mental alienation will remain the general term; I shall also employ the word phrenopathy; I shall say indifferently, monomania, monophreny, monopathy, to designate partial alteration; but when it is necessary to be exact, I shall say, *mono-melancholia*, *mono-phrenalgia*, *mono-delirium*, and also *polymania*, *polymelancholia*, &c. &c.

### Second Part.

*In what manner Mental Diseases may be classified.*—Method is the key of every study. It is impossible to make any real or rapid progress when we walk without resting-places or sign-posts. Method is generally wanting in the study of mental diseases; extreme confusion reigns throughout the terms, the classification, the exposition of the symptoms, and in the ideas upon the nature of the disease.

*The classical divisions.*—Whilst setting forth the basis of a division and a classification, I wish to show you that mental diseases may present themselves under very simple forms, or combined in the most extraordinary and most complicated manner. We must first of all establish a division under the point of view of the morbid form.

Thus the alienations will be:

1. *Elementary*, that is, simple.
2. *Compound*, formed of several simple elements.

In the point of view of the course they follow, they will be:

1. *Continued.*
2. *Remittent.*
3. *Intermittent.*
4. *Periodical.*

With regard to the morbid transformations:

1. *Primary.*
2. *Secondary.*
3. *Transitory.*
4. *Permanent.*

As to their seat:

1. *Idiopathic.*
2. *Sympathetic.*

As to their pathogenic value:

1. *Essential.*
2. *Symptomatic.*

*Elementary forms.* It is the same with mental affections as with every other disease; every case does not represent a case identically analogous. But in mental diseases more than in any other varieties they are multiplied and complicated. I do not think I deceive myself in estimating at more than one hundred, the different forms under which the phrenopathies may present themselves.

The art of observation must be directed to discover in this prodigious number of manifestations the elementary types, the fundamental expressions. Let us borrow a comparison from the musical art. In music, as in language, all the intonations are reduced to a series of fundamental sounds: these are, in music, the seven notes, in language, the five or six vowels. It is the same as in painting, where all is reduced to the prismatic colours.

So it is in diseases, and especially in mental diseases. There are fundamental tones and colours. Alienation has its elementary accords, phrases, words, and colours.

Thus, in order to be able to establish the capital forms under which mental alienation is observed, we must seek for the fundamental characters of the morbid expression.

These characters I find in the six following physiological manifestations:—

A.—A mother seated at the bedside of her child dangerously ill: she is the image of sorrow.

B.—The man, but little accustomed to the attractions of the higher regions of society, struck dumb, stupefied before a prince whom he ought to address: he expresses the characters of stupefaction, of perplexity.

C.—The man who is excited, reacts, becomes angry, defends himself, and exhibits a struggle of words and actions: he represents a mind in a state of exaltation.

D.—He who affects a ridiculous attire, who announces himself everywhere as a man eccentric in his tastes and his conduct, represents singularity of impulse.

E.—Error is found in the projector, in the builder of castles in the air.

F.—Nullity is met with in the creature who is called a simpleton, an imbecile.

It is, therefore, in these groups, taken from the natural condition, that I look for the types of my classification of mental affections. This consists in the six following elementary forms:—

I.—*Melancholia*—Phrenalgia:—exaltation of the sentiment of sorrow.

II.—*Ecstasy*—Phrenoplexy:—suspension of the intellectual acts with general rigidity.

III.—*Mania*—Hyperphreny:—impassioned moral exaltation.

IV.—*Insanity*—Paraphreny:—anomalies of the impulsive will.

V.—*Delirium*—Ideophreny :—anomalies in the ideas.
VI.—*Dementia*—Aphreny :—decadence, obliteration of the moral and intellectual acts.

Each of these forms may present itself either in the simple or compound state. In the simple state, it constitutes a monomania, a monophreny. There are therefore as many monomanias as there are elementary forms of mental alienation.

There is nothing more confused than the denomination of the partial alienations which Esquirol has called monomanias; Marc has already observed this, and it is a matter for surprise that hitherto no one has endeavoured to dispel this confusion.

Monophrenopathy is for us an elementary form, simple, isolated, partial.

*Compound forms.*—In another category we range compound forms, binary, tertiary, quaternary, and yet more complex. These are mixed forms—the *morbi mentis mixti*, designated by Heinroth as follows: polyphrenopathics, polymelancholies, polymanias, polydeliria, &c. &c.

You must well understand the mosaic of symptoms. You will see alienation constantly assuming new forms; now fugitive, now permanent, now simple, now combined in the most fantastical manner. You will meet with sorrow and exasperation, exasperation and eccentricity; eccentricity and error. You will meet with sorrow and error associated with anger; or anger, nullity and delirium, up to the most complex associations.

*Radical phenomenon.*—Art consists in searching in a given group of symptoms for the governing principle, and in pointing out its associated phenomena. Thus I propose to say—

Melancholic mania, if the second form predominates over the others in importance;

Maniacal melancholy, if phrenalgia is the most prominent symptom.

We shall sometimes say delirious mania, and sometimes maniacal delirium; mania with dementia, or dementia with mania, and so on. I find, therefore, in alienation, symptoms that are essential, simple; I also discover symptoms that are secondary. The first designation characterises the genus and leading form of the disease. The others are, in some sort, accessory; they appear and disappear in the course of the disease.

*Proportion of different forms.*—Let us conclude this introduction by a remark relative to the proportion in which the forms of mental alienation present themselves. And, first, I will remark that the frequency of the manifestation of one form or of another varies considerably, according to the different circumstances in operation at the period of its development. Hence, the great difference observed between public and private asylums. The manners of a country, and atmospheric conditions also, influence remarkably the forms of mental diseases. At Ghent we have observed, within these last few years, when the labouring classes have been subjected to the greatest privations, a great increase in the number of demented; this figure has been progressive for two years, so that the morbid forms have exhibited the following proportions:—

Out of 100 admissions there were:—32 cases of dementia; 28 mania; 17 melancholy; 20 incoherence; 2 ecstasy.

Moreover, during the series of years preceding the disastrous years 1847, 1848, and 1850, our public establishments at Ghent contained:

Out of 100 admissions:—35 cases of mania; 25 melancholy; 20 dementia; 20 incoherence; 2 ecstasy.

In the York Retreat, Dr. Thurnam reports the following proportions in 100 admissions:—45 mania; 35 melancholy; 10 monomania; 8 dementia.

At Turin, the tables of Dr. Bonacossa show:—1 maniac in $4\frac{1}{2}$ admissions; 1 melancholic in 4; 1 demented in 5.

Our figures, therefore, approach the normal proportion ascertained in the north of Italy. In the York Retreat, as in our private asylums, the patients do not belong to the indigent class. A greater number of cases of dementia is received into public institutions, always excepting one form, that of general paralysis, which is extremely common in all the private asylums.

In the calculations of M. Parchappe, relating to the asylum of Rouen, we find out of 100 admissions 42 cases of mania; 25 melancholy.

Whereas, mania constituted formerly the chief figure in our numerical statements, it is now dementia. But since the return of prosperity, the forms of alienation are beginning to change, and we are coming back insensibly to the normal figure of former times.

It is, therefore, mania which is the most common form of insanity; melancholy holds the next place; dementia the third, delirium is no longer a frequent disease; ecstasy is very rare.

### Fifth Lecture.

*Exposition of the phenomena proper to the different forms of melancholy.*

### First Part.

I shall make two groups of the phenomena which constitute melancholy. In the one I shall place the melancholy which I shall designate as general; in the other, I shall include the forms of special melancholy. It is to these last that the name of monomania has been given. For us these affections will be *monomelancholia*, *monophrenalgia*. The term *polymelancholia* will designate general melancholy. Every melancholy expresses the lesion of a sentiment; it is a painful affection.

The melancholy may be a grief: that of a wife for the death of her husband. It may be an anxiety: the feeling of a person alienated in consequence of a reverse of fortune. It may be a fear: the dread of having offended God.

Jealousy, envy, horror, do not belong to melancholy, but are met with in other kinds of phrenopathies.

General Melancholy.—In the study of melancholy, whether general or special, we must proceed with order, interrogate each faculty, inquire of each intellectual function the nature of the perturbation they suffer.

First, we address ourselves to the moral element. We pursue the irradiations of the disease into the domain of the intelligence: we study its external manifestations.

*Practical view of a series of melancholic patients.*—In the persons whom I have brought before you, melancholy betrays itself, in the features, in the actions, in the tone of the voice. Everything that these patients answer to your questions will exhibit the tone, the complexion of melancholy; every word they utter will bear the stamp of moral suffering.

These patients accuse themselves. They ought, say they, to have done this; they ought to have done that. One says, I have offended God; another pretends that he has signed a deed compromising his fortune or the fortunes of his children. He knows not what to do; a state of irresolution afflicts him.

This other patient is a prey to gloomy forebodings: he is about to be imprisoned. I no longer love my children, exclaims a mother. I no longer love my husband, says a wife. I no longer pray, says another, I do not love God.

Of all the forms of alienation, melancholy is the most readily converted into religious feelings.

In spite of the grief which overwhelms these patients, they rarely weep. One of them sometimes howls, but he never sheds a tear. In some exceptional cases, melancholics cry, and then they shed torrents of tears; for months together they weep without ceasing.

This condition reacts upon the intelligence, which is in a state of obumbration. The melancholic does not understand, or understands but badly what is said to him. This patient appears to be deaf, although she is not so in reality.

It is especially when the phrenalgia is simple, when it is not associated with other elementary forms of mental disorder, especially to mania, that the muscular system is found to be in a state of prostration. Observe that woman: she is continually sitting; her head is lightly resting on the breast; her eyelids half conceal the eyes. Throughout the day this patient never changes her attitude or her position. It would seem that the influx of the medulla spinalis, medulla oblongata, and cerebral centres, is cut off in its course: the muscular immobility is at the same time accompanied by a slight tension, which is seen in the flexor muscles. Have you well observed the words of this insane woman: "It is of no use to will, I cannot do it. I cannot get up. I can take no resolution?" In fact she has no initiative; it is vain to set her to read, or to do any manual labour; the book, the work, fall from her hands.

In some cases melancholics tell you that they feel in the cranium, or under the scalp, a creeping or prickling sensation: a similar sensation is sometimes referred to the legs and arms.

In some cases they experience frontal or occipital cephalalgia, especially during the onset of the disease.

The condition of melancholy destroys the manifestations of instinct. The patient ceases to be affected by the cold or heat; he would let himself be frozen in the depth of winter; set in the full sun, he would not move. He neglects himself utterly; he does not comb his hair, or wash; he will scarcely eat or drink unless a friendly hand compel him.

On his convalescence, the patient will tell you that he has passed nights without sleeping, that he suffered from pain in the head, and that during his illness he seemed to have no head.

Observe the skin of this melancholic patient: its tinge is brownish or blue; and do not forget that this is not the natural hue of this woman's skin. As soon as convalescence approaches, you will see her skin become clearer, more transparent, and this dusky shade will completely vanish. It is this colour which made the ancients imagine that in melancholy a black bile was mingled with the blood.

Often the lips are bluish. This kind of cyanosis, which you will often perceive in cases of morbid sorrow is, in my opinion, the result of a disorder in the elaboration and circulation of the blood. I regard this complexion as arising from a venous congestion, an imperfect hæmatosis. This condition is easily understood in phrenalgic patients; it is explained by the preliminary prostration, by the feebleness of the mechanical phenomena of respiration. Crouched up, inanimate, they breathe but imperfectly; the inspiratory muscles hardly act. The heart has lost its strength, as well as the diaphragm; these muscles are in the same condition as the locomotive muscles, they are in a state of torpor. It is the same in typhus, as I have proved elsewhere. It is the feebleness of the heart added to the pectoral prostration, the diminution of the quantity of air entering into the chest which produces a stasis of the venous system, and gives to the skin a remarkable leaden hue.

The central organ of the circulation deserves special attention. The impression which has struck the moral element has been reflected upon the heart; the result is frequently two diseases which may present themselves together. Thus this woman before you has her hands constantly of a deep-blue colour, as if she was taken with cholera. Her lips are cyanosed; her nose and ears are livid. There is evidently a disturbance in the functions of the heart, a disturbance which may, perhaps, be nervous, but which may be an organic condition. The death of her child has thrown this unhappy woman into this afflicting state.

Generally in melancholy the skin is cold, unless the patient is well covered in bed.

Examine the pulse: you will find it accelerated; I say accelerated, in order not to confound it with the frequent pulse belonging to febrile diseases. This acceleration of the action of the heart is not, however, a general phenomenon. Not unfrequently the pulse is exceedingly slow. It is seldom full or hard.

I have not yet been able to explain the relations which may exist between this variation of the pulse and the phrenopathic symptoms. I think, however, that I have remarked that the pulse is particularly frequent so long as the patient suffers, and is sorrowful, and that the pulse becomes slow when the disease cuts off the intellectual faculties.

The patient is in the condition of the apoplectic or the hydrocephalic, in whom the circulation is most frequently slow, because the brain ceases to influence the viscera, as is observed in sleep, which is always attended by a falling in the frequency of the pulse.

If you carry your investigations further, you will find that there is scarcely a function which does not undergo a sensible perturbation under the influence of morbid grief. Thus we remark a general diminution in the secretions; the fatty product diminishes everywhere; in a few days the patient has become thin, his skin is dry, his hair even shrivels, the alvine evacuations are sluggish, the secretion of tears is sometimes suppressed, there are obstinate constipations, and sometimes the stools are tinged with dark bile. Nine times in ten the menstrual elimination does not take place.

*Second Part.—Special melancholies.*

The most simple condition under which special melancholy can present itself is:—

I. *The melancholy without delirium* of Etmuller. It is found in the mental affections designated under the names of

*Moral melancholy.*
*Affective monomania of Esquirol.*
*Reasoning lypemania of the same.*
*Melancholia simplex of Heinroth.*

We will keep to the first denomination, saying sometimes melancholy without delirium, and sometimes phrenalgia without delirium. The melancholy without delirium is found in the forms which English authors comprise under the name of *moral insanity.* I estimate that phrenalgia without delirious ideas, presents itself in one half of the cases of melancholy. But of one hundred admissions at Ghent it occurs about thirteen times.

This vesania is exclusively an exaggeration of the affective sentiments; it is in all the force of the term a *Gemüthskrankheit* in the sense employed by the Germans. It is a pathological emotion, a grief, a sorrow, an anxiety, a dread and nothing more. It is not a condition which sensibly affects the conceptions. Nor is it a condition in which the patient presents any remarkable anomalies in the actions.

This vesania may constitute the incubation stage of a more serious condition. It may also constitute the terminal period of other mental affections.

Nothing astonishes more than these men, profoundly afflicted, who analyze all their ideas, all the phenomena of their morbid condition; who reason with perfect lucidity of conscience concerning the impotence of their will, upon the ardent desire they experience to escape from this state of dread and wretchedness. Thus, the other day one of my melancholic patients who had been cured, experienced a relapse, and said to me: I don't think my cure was real, for the situation in which I found myself was one of exaltation; I got up too early in the morning; my sleep was disturbed; I had too much activity, and now I have too little. I could wish to be always in bed; the whole of my body does not seem to belong to me.

The knowledge of these morbid shades is of great importance when, as in medico-legal cases, it is necessary to decide whether the patient be or not responsible for his actions. A few days ago a lady came to consult me, saying: "You see before you a person who knows perfectly well what she says, does, and thinks; but I am overwhelmed with an uncontrollable sadness. In society I can overcome this melancholy for a few hours. When alone I abandon myself to the most frenzied excesses. And yet I am a happy woman; I love my husband and my children, but I carry in my heart a pain, an agitation which do not leave me a moment's repose."

The appreciation of this state is of importance in another respect, that of the deductions which it may supply as regards the prognosis. We shall find that the further melancholy departs from the type of its fundamental alteration, the less favourable are the chances of cure.

Many mental physicians, especially in our day, have passed over in silence this remarkable variety of melancholy, which is characterised by the absence of delirious ideas. From the time of Pinel it has been said that melancholy consists in the extreme intensity of an exclusive delirium; it is insisted upon, that there exists in this affection a certain appreciable disorder in the conceptions. Lorry, however, had perfectly made known the *melancholia sine delirio*, in opposing the opinion of Boerhaave, who saw nothing in this affection but delirious ideas. "*Non enim omnes deliri dici possunt*," says Lorry, "*qui timore aut mœstitia præter rationem afficiuntur et melancholico morbo laborant.*"

Every day I meet with melancholics who manifest no disorder in the ideas, or in the faculties of appreciation. It is true, it has been thought proper to exclude these affections from the list of mental diseases; but this is wrong.

Thus, Fred. Nasse does not think it right to include the simple lesions of sentiment in the number of true alienations. In his work, *Die Regelwidrigkeiten der Gefühle*, this phrenographer has developed at great length a view which I cannot adopt.

Several affections, characterised by sadness without delirium, may put on the monophrenic form. We find among others, hypochondriac melancholy, nostalgia, erotic melancholy, and misanthropic melancholy. These affections are often also ordinary phenomena of polymelancholia. They may be permanent phenomena or transitory symptoms.

II.—Melancholy is sometimes characterised by an extreme uneasiness as to health. The patient has a constant propensity to occupy himself about ailments which are seldom real. This is the *hypochondriacal melancholy* of Sennert, the *cerebropathy* of Georget, the *cerebro-ganglionic morotaxy* of Brachet, the *hypochondriacal monomania* of Dubois d'Amiens, the hypochondria of the majority of authors.

This affection might be more properly called *pathophobia*, or *monopathophobia*. It is the condition to which the vulgar frequently give the name of *nervous affection*. It must be regarded as one of the lightest shades of the phrenopathic state, and for this reason it belongs of right, as I have just said, to moral vesania. This disease holds a doubtful place in nosographical arrangements. Some regard it as a true alienation, others class it among the neuroses, and compare it to hysteria. But hypochondria is a disorder of the moral element, and certainly an alienation. What proves it, are the transformations of this affection into other mental diseases.

Hypochondria presents two forms which I wish to describe to you: the first is the condition which I shall name *corporal hypochondria;* the other is *mental hypochondria*, hypochondriacal melancholy properly so called.

Those who are the subjects of the variety of corporal hypochondria say they are ill, and suffering. They believe they have every disease, every infirmity; they feel all the ailments they hear spoken of. They apply to physicians, amateur doctors, apothecaries, and quacks, with the object of explaining to them their disease, and of seeking remedies, which they mostly take with avidity.

Corporal hypochondria is a rare disease here. It does not occur once in two hundred admissions. It is more frequently met with in the world.

Hypochondriacs do not come into asylums until a very advanced period of their disease.

In mental hypochondria there is a different *facies*, there is an expression of a more abstract sensation, more essentially melancholic; there is a phrenopathic shade more strongly marked.

This is a state in which the patient examines himself, and experiences a continual desire to speak about all his moral sufferings. Nothing is so painful to this patient as the perception that no attention is paid to his complaints. Sadness is the prevailing phenomenon of this disease, but it is always a fear, a dread. "If I had done this," he says; "if I had done that! I have neglected to seek you; my whole economy is deranged; I have lost my health."

The hypochondriac experiences the strangest symptoms, he complains of vertigo, of a void in the cranium, of unfitness for all intellectual exertion; he exhibits a great impressionability of the senses; he puts unlimited and absurd confidence in such and such a substance. A dread of going out, of exposing himself to the air, oppresses him.

Hypochondria is often symptomatic. It accompanies neurosis of the heart, affections of the pericardium; it is connected also with gouty disposition; it attends involuntarily spermatorrhœa in persons who have arrived at a certain age.

It is not at all rare in abdominal obesity, and as a general rule, it affects the strong and sanguineous as well as the spare, delicate, and nervous.

The critical age in women is a source of hypochondriacal melancholy. They weep incessantly, complain of intolerable pain without being able to point out where they suffer. They are tormented with fears and vague alarms as to the state of their organs. This condition is attended by drawing in of the abdominal walls, emaciation, and constriction of the throat.

Hypochondriacal melancholy is in its nature very chronic; often it is accompanied by a visible dilapidation of the physical health. The patient has a sallow complexion, rings round the eyes; he is constipated, and troubled with eructations; he has palpitations; pain and uneasiness in the region of the liver, and spleen; cardialgia, a fantastic appetite; his stomach sinks in, or becomes developed and hard to the touch. It is not rare to find an hæmorrhoidal flux, or a vomiting of black blood.

Hypochondriasis may show itself either in the simple state, or in the state of complex alienation. It may be present as a predisposing element to mental diseases. It may constitute the prodromic period of other mental affections; it may be a true moral insanity, and last a long time before assuming the character of a well-marked morbid condition.

Hypochondriasis may undergo different transformations. It is not rare to see it metamorphosed into religious melancholy; the alarms of the patient are converted into ideas of despair; these in their turn are transformed into delirious ideas, into conceptions relating to eternal punishment, into demonophobia. Frequently enough, also, suicidal ideas arise. There are cases in which we see it complicated with mania. It may constitute the intercurrent period of two attacks of mania, or of intermittent or periodical melancholy. It may appear as a character of an imperfect convalescence from mania, or general or special melancholy.

*Nostalgia.*—In Belgium, at the present time, we have no opportunity of witnessing this affection.

*Eroto-melancholia*, amorous melancholy, is a rare affection. Many cases of melancholy take their rise from an unhappy attachment, but there are few in which the amorous feelings are preserved. It is not seen more than once in four hundred admissions, at least here. It may, indeed, constitute the prodromic period of erotomania. It has generally been confounded with this last, which is, however, a very distinct affection.

A *misanthropical melancholy* has been described (*Melancholia misanthropica*, of Sauvages; *antipathica*, of Heinroth). Those afflicted with this form seek for solitude, and shun the contact of men. They withdraw themselves into out-of-the-way places, sometimes behind furniture, bales of goods, or rubbish; and there they will remain for days together without eating or drinking.

Misanthropical melancholy, in the simple condition, is a rare disease. In every case, aversion for society, the longing for solitude, repugnance for the pleasures of the world, are of the essence of every melancholy. This form of alienation is often the forerunner of religious melancholy, of suicide, and of homicide.

This vesania must not be confounded with the physiological misanthropy, which is that within persons under the influence of a great sorrow. Nor must it be confounded with that state which is often associated with religious ideas, and which determines certain persons to quit the world, to live in solitude, and to devote themselves in a convent to the exercise of religion, and meditation upon the greatness of God.

*A patient affected with anxious melancholy.*—Look at this patient: her pupils are dilated, a characteristic pallor is spread over her features. This woman often throws her head back, sighs, and sobs; she is agitated; she is, she says, *hunted.*

This is the melancholy which I call *anxious*, or *pneumo-melancholia*, on account of the disorder which reigns in the organs of the chest. The agonies which the patient experiences sometimes resemble fits of suffocation. Sometimes this is allied to hysteria; more frequently it is independent. Anxious melancholy is sometimes preceded by a painful sensation which the patient refers to the region of the heart. This may last for two or three months before a decided mental state breaks out. The patient becomes sleepless. Gloomy ideas besiege him. His features lose their wonted expression. Attacks accompanied by vague terrors announce the onset of the disease.

This variety of melancholy, in some cases, hardly oversteps the proportions of a moral insanity. It is then free from all disorder of the intelligence, and the patient is constantly saying to those who possess his confidence, that he is afraid of going out of his mind. I have known patients continue two or three years in this situation, without ever betraying the slightest derangement in the intelligence, and still less in the ideas.

Sometimes the pulse is extremely frequent and feeble, and sometimes not greatly disturbed; the skin preserves its ordinary temperature; the sleep is tolerably good; sometimes the appetite is indifferent.

The person we are now examining is astonished at her own situation; she is afraid of it; "I know not what I am doing," she says; "I feel myself capable of committing a crime, I am good for nothing, I think I shall choke." Her attacks are sometimes manifested suddenly; they compel her to toss about in every direction. Fifty times in succession she makes the tour of her apartment, or of the yard. She pronounces the name of a person or an object; she laments, her ideas become confused, and she acts at hazard. This state comes on in fits; each fit may last but for a few hours, but it may last for days and weeks.

Anxious melancholy may be the precursor of an attack of epilepsy. It constitutes the prodromic period of suicidal insanity. It is pretty frequent in women at the critical period.

*Flemming* has lately given to this state the denomination of præcordial anxiety, *Præcordialangst.*

*Three patients afflicted with religious melancholy. Religious monomania, religious monomelancholy.*

During the years of calamity which we have lately passed through, religious fears have borne upon our tables of admission, the proportion of 0·58 to melancholy.

The first of these patients especially dreads the flames of hell. This is the *demonomania* of Sauvages. I call it *demonophobia*, *monodemonophobia*.

The word mania has been very improperly imported into the designation of this disease. It is essentially a phrenalgia, a melancholy, and above all a fear; it is the flames of hell which terrify the patient. There is, as I shall explain in speaking of delirium, a variation of this affection in which the patient thinks he sees flames and conflagrations everywhere around him.

These morbid fears arise from excessive religious fervour, the abuse of religious exercises, great misfortunes which concentrate all the feelings, all the ideas upon religious hopes, exaggerated fears relating to the torments of hell, too frequent confessions, missions, religious festivals.

Demonophobia may take the epidemic form.

A distinction must be drawn between what I call demonophobia and demonolatry. In demonophobia the patient is under the dominion of continual fear; his future fate incessantly haunts him, he exaggerates beyond all bounds his real or imaginary faults.

In demonolatry, the disease has another appearance. The subject believes himself possessed by the demon, and yields him worship. He gives himself up with a satanic pleasure to the illusions of his imagination.

*Melancholia desperatoria* is a form which melancholy occasionally assumes.

*Compound melancholies.—A patient affected with melancholy and mania.*— From the preceding morbid forms, there often arises the *mania melancholica* of Lorry, the *tristomania* of Rush, *maniacal melancholy.*

This woman presents, in the phenomena of her disease, a mixture of actions, belonging on the one hand to melancholy, and on the other to mania. Her countenance is expressive of sadness; her cheeks are inundated with tears; her speech reveals painful thoughts. But she is standing; her eyes are open; her look is bold; she will not bear contradiction. Her behaviour is aggressive; it is often necessary to isolate her. Her grief bears a character of extravagance. She eats well, and even much. The skin is hot, the pulse frequent.

You perceive that sadness characterises the disease, but that there is also present an element of activity, of reaction. Mania is associated with the melancholy.

Maniacal melancholy may often exist with complete integrity of the intellectual functions. Some of these patients reason with clearness, and analyze all the phenomena of their disease:—"I am calm at present, they say, but wait, my sufferings will soon return; I shall no longer be master of myself; I shall not be able to prevent myself from crying out, from shrieking and frightening every body."

I have observed melancholy alternating with mania; at other times I have observed a complete fusion between these two phenomena, comprising at the same time sadness and violence. I have at this moment under my care a patient who is melancholic every fourth day, and maniacal for the rest of the time.

This next patient presents a complete mixture of the two orders of phenomena; he sobs, talks, and shows at the same time a strong propensity to anger.

The ancients understood these combined conditions better than we do: often they have included under the same denomination, melancholy and mania. According to them melancholy arrived at an advanced stage, always constitutes a mania.

(Aretæus, Cælius Aurelianus, Alexander de Tralles, Van Lom, Marchand, Manet, Boerhaave, all express this opinion.)

This idea, which establishes an alliance between melancholy and mania, after having endured through centuries, appears to end with Willis, who alone, among the moderns, has borne it in his mind. In his later writings, indeed, Esquirol has spoken of a *melancholic mania.*

I have thought it necessary to give to one of the varieties of maniacal melancholy, the name of *Rabies melancholica*, to designate that condition of phrenalgia which exhibits all the characters of despair, carried to a veritable fury.

*Suicidal melancholy* most frequently arises out of a combination of elementary forms.

*Homicidal melancholy* arises in a similar manner.

In these forms of melancholy we often witness refusal of food. I have given to this serious symptom the name of *Sitophobia* from *Sitos*, food, and *phobos*, dread.

Compound melancholy is sometimes formed exclusively of an union of sadness with delirious conceptions. Patients believe themselves doomed to the guillotine; others from religious motives, think they are compelled to immolate their children. This is the *melancholy with delirium.*

*The phases and causes of melancholy.*—We may recognise the phenomena of incubation, the initial phenomena or those of invasion, the phenomena of morbid progression, stationary phenomena, the phenomena of morbid retrogression, and those of convalescence. We may also observe phenomena, announcing the transformations of the disease.

Most frequently, melancholy presents itself as an initial primitive vesania, as an elementary phrenopathic lesion. It may also be a transitory apparition, arising in the course of other mental alienations. Phrenalgia is, in certain cases the terminal phenomena of some other mental disease. To the observant physician it is often the index of approaching convalescence, when this disease is developed slowly in the course of mania, and at that epoch when the patient has passed the stationary period.

Melancholy rarely breaks out suddenly. I have, however, occasionally witnessed cases in which the disease had begun by a kind of commotion, as if by little blows felt in the cranium. I have sometimes observed as a phenomenon of invasion, attacks resembling hysteria.

One of the first symptoms is the loss of sleep. The patient is pursued by sinister ideas. His head seems on fire; his features change; his eye is dull; the man has grown old. He forgets his duties, neglects his business; he is an altered man. Every one perceives the change which has taken place in his physiognomy. And yet up to this point, he is in no one's eyes a madman. He makes great efforts to ward off his affliction; he would think of anything else, but cannot. Nothing interests him; he seeks to be alone; he no longer speaks to his wife or children. Morbid melancholy reveals itself. The course of this affection is at first slow and interrupted. After a few days some alleviation is observed; everybody is rejoiced, and the inexperienced physician gives hopes of recovery. But throughout the whole period of the morbid progression these symptoms are treacherous.

To this calm an aggravation succeeds, then a relief of short duration. The patient has his good and bad days, until the melancholy, becoming more and more severe, presents no intermission or remission. Let us add, that there is a kind of diurnal oscillation; in the morning there is great oppression, in the evening more clearness and calm. But this rule is not general.

When the disease has reached its stationary condition, it may no longer vary in its form. It may remain simple, without delirium, throughout, or, from simple it may become complicated with delirious ideas; it may assume the religious form or the suicidal.

If the melancholy remain in the initial shade, if it be a first invasion, and the subject young—the disease runs its course in three, seven, or nine months. It may happen that recovery will not take place under three, four, five, or six years, but this is rare. Sudden cures are in frequent, and apparently rapid amelioration deceitful. In a good cure the morbid elements exhaust themselves slowly.

The indications of future amelioration consist in moments of calm; the patient's features are strikingly changed; they acquire animation, and one is astonished to hear him speaking like a person of a sound mind. At first there are but flickerings of moral liberty; then these lights of the intelligence become more constant, and last for half an hour or an hour. They return in a few days, and constitute true lucid intervals which become longer and longer.

In other cases, however, the cure is laborious; this situation may even end in a relapse.

Sometimes during convalescence the melancholy has disappeared during the day and returns at night. This nocturnal return is not peculiar to melancholy, but is observed in mania and other forms of vesania.

As to the prognosis, I may say that, if properly treated, seven-tenths of melancholic patients recover.

If melancholic convalescents be ever so little excited, they evince a propensity to laugh immoderately. They like to dress themselves, and to chat; their form denotes a mobility which contrasts with their previous state; they seek places of public resort. This state of gaiety calls for management; it vanishes in a few weeks. It seems as if, during this transition from sickness to health, something suddenly breaks into the intellectual domain and excites it.

In certain cases this exultation bodes no good. It leads to a return of melancholy, or some other form of disease. In these cases the patient shews irritation in his features. His eye opens; he no longer sits down; he questions, talks, declaims, comes and goes; he is dissatisfied, and complains; he wishes to go away; he meets enemies everywhere about him. In a few days an attack of mania breaks out. Sometimes the phrenalgic condition alternates with these attacks.

The disease may become essentially chronic. In such a case, prostration ceases; the patient recovers a certain aptness for work, and suppleness in his limbs. The pulse becomes normal; he recovers sleep, and freshness of skin; but sadness persists.

When melancholy occurs in aged, broken-down people, it takes an atonic form, and may constitute an incurable affection.

The passage of melancholy to an incurable chronic state, is often announced by a marked relaxation of the muscles of the face, a change in the face, absolute negligence in attire, indifference for everything. In recent cases, however, these phenomena are without value.

In a few cases, abdominal marasmus carries the patient to the grave. This condition is connected with visceral engorgements. It is characterised by hardness and swelling of the belly, by habitual constipation, and a darkened complexion. At the same time the patient grows very thin.

Rarely melancholics succumb to cerebral symptoms, announcing the existence of organic alteration.

It happens sometimes that they die suddenly without apparent cause. Such a termination most usually occurs at an early period of the disease.

Suicide may be the terminal phenomenon.

